# Highly Ordered Bimodal Mesoporous Carbon from ABC Triblock Terpolymers with Phenolic Resol

**DOI:** 10.1021/acsmacrolett.4c00651

**Published:** 2024-12-05

**Authors:** Yuta Miyamori, Youngwon Kong, Yuta Nabae, Kan Hatakeyama-Sato, Teruaki Hayakawa

**Affiliations:** Department of Materials Science and Engineering, School of Materials and Chemical Technology, Institute of Science Tokyo, 2-12-1 S8-36 Ookayama, Meguro-ku, Tokyo 152-8552, Japan

## Abstract

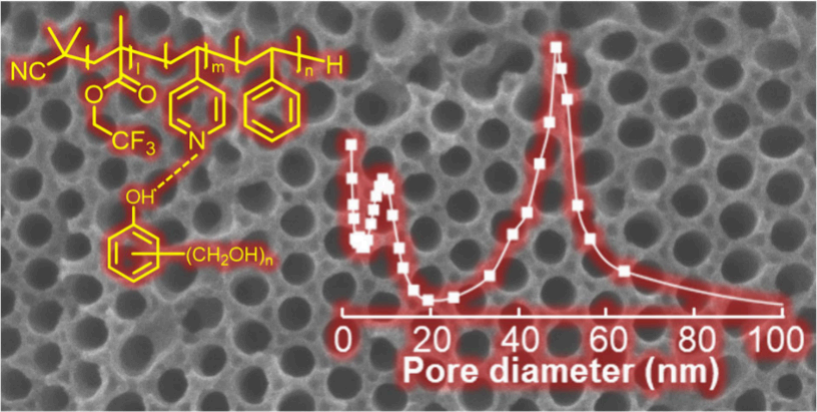

Mesoporous carbons (MPCs) with a bimodal distribution of pore diameters are more advantageous than their monomodal counterparts for applications in adsorption, catalysis, and drug delivery systems; however, reports on their fabrication remain limited. In this study, we successfully fabricated bimodal MPCs using a soft template method with poly(2,2,2-trifluoroethyl methacrylate) (PTFEMA)-*b*-poly(4-vinylpyridine) (P4VP)-*b*-polystyrene (PS) and resol. The blend samples formed microphase-separated structures comprising PTFEMA spheres, PS cylinders, and matrix domains composed of P4VP and resol, leading to the separation of the PTFEMA and PS domains. The P4VP and resol matrix domains were carbonized at a high temperature of 900 °C, whereas the PTFEMA and PS domains were thermally decomposed. This process resulted in bimodal MPCs with both spherical and cylindrical mesopores. The pore diameters calculated using scanning electron microscopy were approximately 10 and 30 nm, while nitrogen adsorption measurements indicated a large specific surface area with a bimodal pore distribution.

Highly ordered mesoporous carbons (MPCs) are attractive for applications in adsorption,^1−3^ separation,^4,5^ electrode design,^6^ drug delivery,^7,8^ and catalysis^9^ owing to their high specific surface areas, large pore volumes, and superior thermal, mechanical, and chemical stability. Porous carbons (PCs) with a bimodal distribution of pore diameters, referred to as bimodal PCs, are expected to exhibit higher potential for adsorption, catalysis, and drug delivery than monomodal PCs.^10−14^ Guo et al. reported the fabrication of bimodal PCs with micro- and mesopores.^10^ Mesoporous films were produced by soaking polystyrene (PS)-*b*-poly(2-vinylpyridine) (P2VP) in hot ethanol. These films were filled with resol, followed by cross-linking, carbonization, and pyrolysis of the block polymers, resulting in bimodal PCs that exhibited excellent electrochemical performances. Similarly, Zhao et al. successfully fabricated bimodal PCs with meso- and macropores. F127 (polypropylene (PPO)-*b*-poly(ethylene oxide) (PEO)-*b*-PPO) and the resol solutions were added to colloidal silica crystals with silica diameters of 240, 320, and 450 nm. The removal of silica particles using hydrofluoric acid after carbonization yielded bimodal PCs with meso- and macropores corresponding to the microphase-separated structures of F127 and the diameters of silica particles.^15^

As mentioned above, although several studies on bimodal PCs have been reported, they are generally limited to cases where the pores are distributed in the micro (approximately 2 nm)–meso (2–50 nm) or meso–macro (50 nm) regions or where the pore structures are disordered. Few studies have described bimodal MPCs with highly ordered pores, particularly within the mesopore region. These bimodal MPCs are expected to be highly effective in the adsorption and separation of large molecules and viruses.

MPCs are primarily fabricated by using either hard or soft template methods. The hard template method can be used to create highly ordered MPCs with various symmetries by using mesoporous inorganic porous materials. However, the hard template method requires the removal of templates using hydrofluoric acid and alkalis, such as sodium hydroxide.^16−21^ In contrast, the soft template method that utilizes block copolymers and cross-linkers is a cleaner approach, as it can create MPCs using only thermal treatment without the need for hydrofluoric acids or alkalis, such as sodium hydroxide.^22−24^

The soft template method facilitates the fabrication of MPCs with various morphologies including three-dimensional network structures from PPO-*b*-PEO-*b*-PPO,^25−27^ PS-*b*-PEO,^26^ PS-*b*-poly(4-vinylpyridine) (P4VP),^28,29^ polydimethylsiloxane (PDMS)-*b*-PEO,^30^ polyisoprene (PI)-*b*-PS-*b*-PEO,^31−33^ PI-*b*-PS-*b*-P4VP,^34,35^ and PEO-*b*-poly(ethyl acrylate) (PEA)-*b*-PS.^36^ However, AB and ABA block copolymers, which are composed of two components, typically form only one type of matrix domain and one type of isolated domain, respectively, yielding MPCs with monomodal mesopores. Similarly, ABC triblock terpolymers composed of three components can form monomodal mesopores when the end-positioned PEO blocks are used, for example, when PI-*b*-PS-*b*-PEO^31−33^ and PEO-*b*-PEA-*b*-PS^36^ are mixed with cross-linkers, such as resol, through hydrogen bonding. In contrast, when a hydrophilic block that can form hydrogen bonds with cross-linkers is positioned as the central B component in the ABC triblock terpolymers, two independent types of mesopores are expected to form, each derived from the A and C domains. Therefore, ABC triblock terpolymers have the potential to fabricate highly ordered bimodal MPCs, particularly in the mesopore region. Although bimodal mesoporous silica has been successfully fabricated using ABC triblock terpolymers with hydrophilic B blocks,^37^ to our knowledge, bimodal MPCs have not yet been acquired.

In this study, we aim to create highly ordered bimodal MPCs using poly(2,2,2-trifluoroethyl methacrylate) (PTFEMA)-*b*-P4VP-*b*-PS (FPS triblock terpolymer) and resol as cross-linkers (Scheme 1). The P4VP blocks located in the middle of the FPS triblock terpolymers interacted with resol through hydrogen bonding, effectively separating the PTFEMA and PS domains. Consequently, after carbonizing the FPS triblock terpolymer and resol blend systems, bimodal MPCs featuring two types of mesopores derived from the minor domains of PTFEMA and PS were expected. The microphase-separated structures of the blend samples before carbonization were characterized by using transmission electron microscopy (TEM) and small-angle X-ray scattering (SAXS). The resulting MPCs were analyzed by using scanning electron microscopy (SEM) and nitrogen adsorption measurements.

**Scheme 1:**
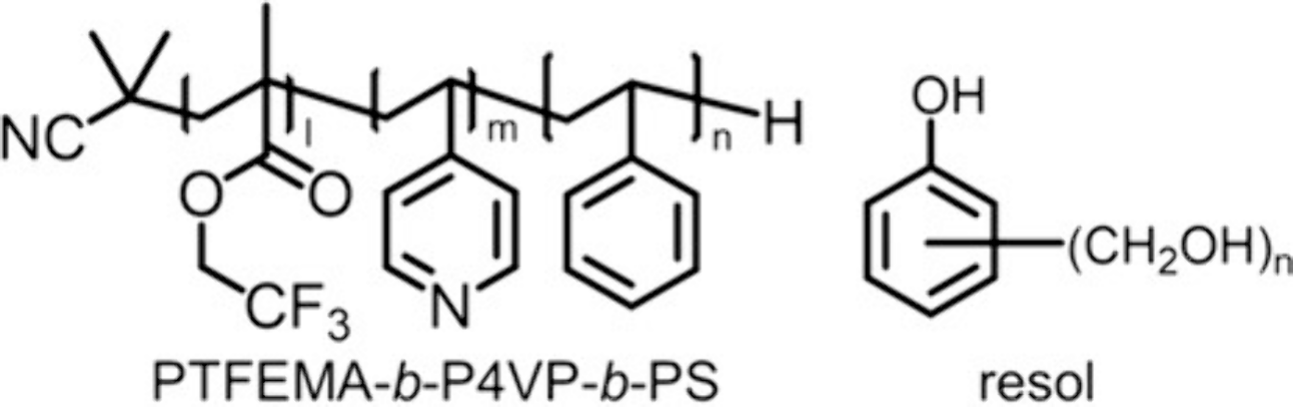
Chemical Structures of FPS Triblock Terpolymer and Resol

Referring to a previous report,^39^ three FPS triblock terpolymers with fixed PTFEMA and P4VP block lengths and various PS block lengths were synthesized via radical addition–fragmentation chain transfer polymerization. Each monomer was sequentially added using 2-cyano-2-propyl dodecyl trithiocarbonate as a chain-transfer agent. The molecular weights of the compositions were calculated from the peak area of each component acquired from ^1^H NMR, and the polydispersity was determined using SEC with 50 mmol L^–1^*N*,*N*-dimethylformamide (DMF) (see Figure S2). Resol was synthesized using phenol and formaldehyde under alkali conditions.^24^

Blend samples prepared using DMF solutions of the FPS triblock terpolymers and resol were named FPS_A_(X/Y), where A is the block length of the PS block, and X/Y is the weight ratio of the FPS triblock terpolymers and resol. Table 1 lists the molecular characteristics and compositions of the FPS triblock terpolymer and blend samples. Furthermore, the blend samples were thermally treated at 900 °C to carbonize the resol-containing domains and remove the PTFEMA and PS domains. The MPC acquired after carbonization included approximately 1% nitrogen atoms and approximately 90% carbon atoms (Table S1). This finding indicated that nitrogen-doped MPCs were acquired. Furthermore, the fluorine concentration was below the detection limit (0.3 wt %) signifying the complete achievement of thermal decomposition of the PTFEMA and PS segments.

**Table 1:** Molecular characteristics and compositions of FPS triblock terpolymers and blend samples

	molecular weights^a^ (*M*_n_; kg mol^–1^)						
sample name	PTFEMA	P4VP	PS	*D*^b^ (−)	*φ*_PTFEMA_^c^	*φ*_P4VP_^c^ or *φ*_(P4VP+resol)_^c^	*φ*_PS_^c^
FPS_48_	13.4	14.4	47.5	1.09	0.14	0.19	0.68
FPS_54_			53.9	1.09	0.13	0.17	0.70
FPS_57_			57.0	1.10	0.13	0.16	0.72
FPS_48_(80/20)			47.5	(−)	0.11	0.33	0.56
FPS_54_(80/20)			53.9	(−)	0.10	0.32	0.58
FPS_57_(80/20)			57.0	(−)	0.10	0.32	0.59

a Determined by ^1^H NMR.

b Determined by SEC.

c Determined by ^1^H NMR, here densities of PTFEMA, P4VP, PS, and resol are 1.45, 1.15, 1.04, and 1.25 g cm^–3^.

Figure 1a–c show TEM images of the blend samples before carbonization and staining with iodine. In these TEM images, the PTFEMA, PS, and P4VP domains appear as the brightest, grayest, and darkest regions, respectively. This contrast arises because the PTFEMA blocks are readily etched by electron beams, whereas the P4VP blocks stain well with iodine. The TEM images show the same types of structures. The left side of the TEM images highlighted a cross-sectional view of the cylindrical domains, and the gray PS cylindrical domains were hexagonally arranged with satellite six-coordinate bright PTFEMA domains. In contrast, the right side of the TEM images shows a side view of cylindrical domains, and the bright PTFEMA spherical domains were aligned along the gray PS cylindrical domains. Furthermore, the 1D SAXS profiles of the blend samples before carbonization exhibited characteristic Bragg peaks corresponding to the square roots of 3 and 7, indicating 6-fold symmetry. These structures consist of hexagonally packed PS cylinders surrounded by six-coordinate PTFEMA spheres. The spherical domains are aligned along the PS cylinders (Figure 1e,f). Furthermore, according to the 1D SAXS profiles (Figure 1d), carbonization changes the primary peaks to higher *q* values. This change was attributed to the thermal shrinkage of structures during carbonization at 900 °C.

**Figure 1:**
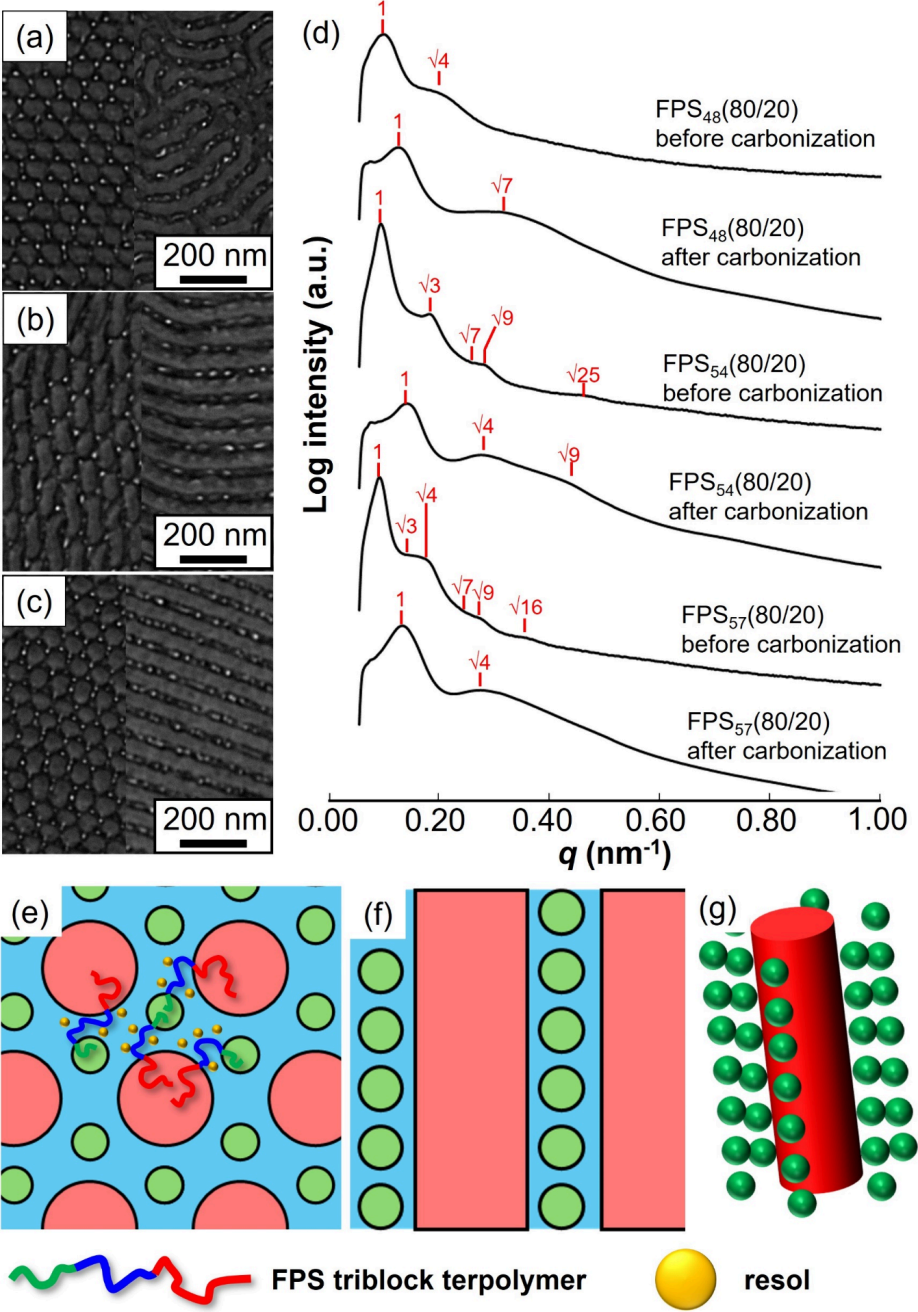
(a–c) Transmission electron microscopy images of FPS_48_(80/20), FPS_54_(80/20), and FPS_57_(80/20) stained with iodine before carbonization. Left and right sides show cross-sectional and side views of cylindrical domains. (d) SAXS profiles (1D) of FPS_48_(80/20), FPS_54_(80/20), and FPS_57_(80/20) before and after carbonization. (e, f) Schematics of microphase-separated structures of blend samples from top and cross-sectional views of cylindrical domains and possible chain conformations in bulk state, where green, blue, and red represent PTFEMA, P4VP, and PS domains, respectively. (g) 3D model of cylindrical PS and spherical P4VP domains.

Generally, in the microphase-separated structures of ABC triblock terpolymers, the component with the highest volume fraction tends to form a matrix domain. Ahn et al. reported a similar structure with cylindrical P2VP and spherical PI domains using PI-*b*-PS-*b*-P2VP (*φ*_PI_:*φ*_PS_:*φ*_P2VP_ = 0.120:0.748:0.132).^38^ However, in the blend samples in this study, PS, despite having the highest volume fraction (0.56, 0.58, and 0.59 in FPS_48_(80/20), FPS_54_(80/20), and FPS_57_(80/20), respectively), forms cylindrical domains rather than matrix domains. The formation of cylindrical domains was attributed to the difference in the affinity of each segment for DMF. PS has a lower affinity for DMF than for PTFEMA and P4VP.^39^ Therefore, in the drying process of the FPS triblock terpolymer DMF solutions, the PS blocks first formed isolated cylindrical domains. The P4VP and PTFEMA blocks then formed a matrix and isolated spherical domains.

Figure 2a–c show the SEM images of MPCs derived from FPS_48_(80/20), FPS_54_(80/20), and FPS_57_(80/20). These SEM images showed the same type of structure. The upper panels of Figure 2a–c show that the large mesopores are hexagonally arranged with six small mesopores surrounding them. In contrast, in the lower panels of Figure 2a–c, which show side views of the cylindrical mesopores, thick stripe patterns corresponding to cylindrical mesopores and spherical mesopores are observed. Cylindrical and spherical mesopores were derived from the PS and PTFEMA domains before carbonization. However, some spherical domains were not perfectly separated owing to the collapse of the carbon walls. Figure 2d shows enlarged images of MPCs derived from FPS_48_(80/20); the upper and lower SEM images show the top and side views of the cylindrical mesopores. The diameters of the cylindrical and spherical mesopores, measured using SEM images, were approximately 30–35 and 10 nm, respectively. Furthermore, the diameters of the cylindrical mesopores of FPS_48_(80/20), FPS_54_(80/20), and FPS_57_(80/20) are 29.1, 30.2, and 32.1 nm based on the primary peaks of the 1D SAXS measurements (Figure 1d), which correspond well with the SEM images. The diameters of the cylindrical and spherical domains of the blend samples before carbonization were approximately 56 and 13 nm, respectively. The SAXS experimental results show an inverse relationship between the primary SAXS peak positions and molecular weights of PS blocks in FPS_48_(80/20), FPS_54_(80/20), and FPS_57_(80/20) prior to carbonization (Figure S3(a)). However, this relationship changes slightly after carbonization (Figure S3(b)), possibly due to the differing ratios of P4VP domains and resol in each blend sample. As described above, carbonization leads to structural shrinkage, which is consistent with the TEM (Figure 1) and SEM (Figure 2) observations. Although the morphology induced by the solvent (Figure 1a–c) may not be thermodynamically stable, cross-linking by resol likely helps preserve this morphology during the drying and heating process at 100 °C for 24 h.

**Figure 2:**
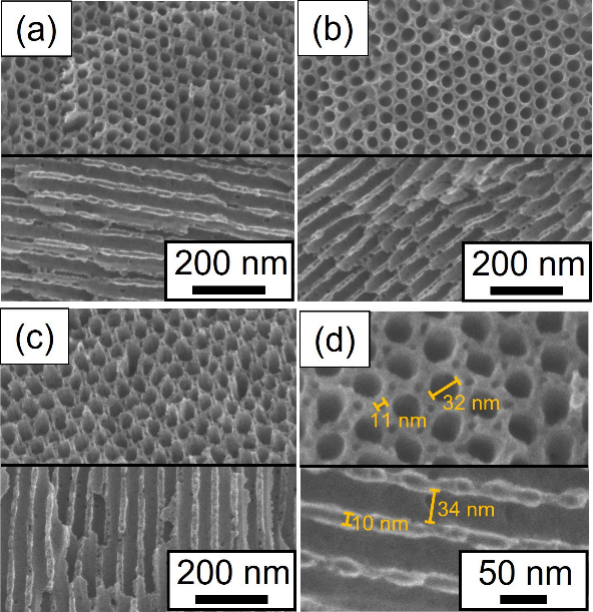
(a–c) Scanning electron microscopy (SEM) images of FPS_48_(80/20), FPS_54_(80/20), and FPS_57_(80/20) after carbonization. (d) Enlarged SEM images of FPS_48_(80/20) after carbonization. Upper and lower SEM images show top and side views of cylindrical mesopores, respectively.

Nitrogen adsorption was measured to analyze the mesopores of the MPCs. MPCs milled using an agate mortar were used in a vacuum after thermal pretreatment at 350 °C for nitrogen adsorption measurements. Figure 3a,b shows the nitrogen sorption isotherms and pore diameter distributions of FPS_48_(80/20), FPS_54_(80/20), and FPS_57_(80/20) after carbonization. The isotherms in Figure 3a–c show typical type-IV curves with HI-type hysteresis exhibiting sharp and blunt capillary condensation above relative pressures of approximately 0.95 and 0.83. Based on the Brunauer–Emmett–Teller theory, the specific surface areas calculated for FPS_48_(80/20), FPS_54_(80/20), and FPS_57_(80/20) were 700, 686, and 699 m^2^ g^–1^, respectively, after carbonization. In contrast, analysis using the Barrett–Joyner–Halenda (BJH) theory resulted in specific surface areas of 179, 168, and 162 m^2^ g^–1^ for FPS_48_(80/20), FPS_54_(80/20), and FPS_57_(80/20) in the mesopore regions after carbonization, with approximately 75% of these specific surface areas attributed to the micropore regions.

**Figure 3:**
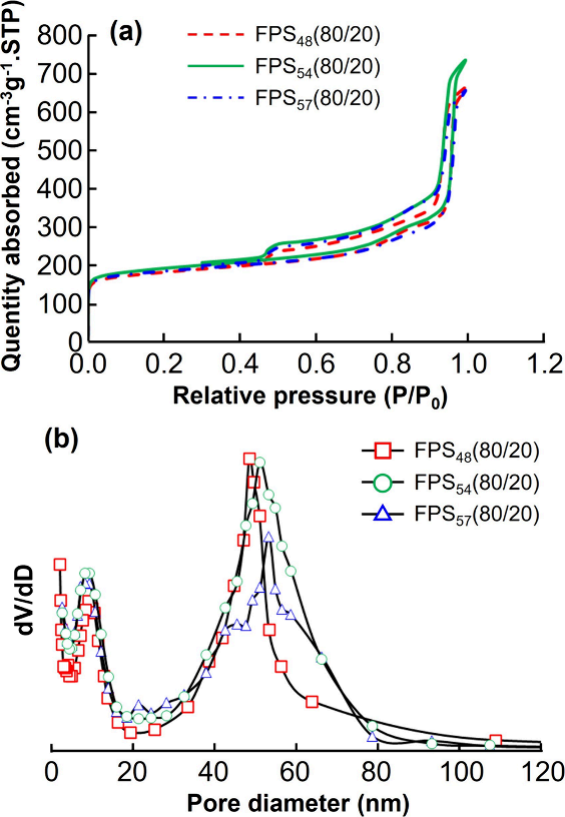
(a) Nitrogen adsorption measurements and (b) pore diameter distributions of FPS_48_(80/20), FPS_54_(80/20), and FPS_57_(80/20) after carbonization.

The pore diameter distributions calculated using the BJH theory (Figure 3b) show a bimodal distribution in the mesopore region for all samples. The graphs show sharp peaks at 9 nm and broad peaks at 50 nm in the diameter position. In Figure 3b, the sharp peaks indicate that the peak tops are located at the same position (9.2 nm) in all the samples, which correspond well with the SEM results (Figure 2). Smaller mesopores were derived from the PTFEMA blocks and had the same diameter, because the molecular weights of the PTFEMA blocks were equal in all samples. In contrast, the broad peaks indicated that the positions of the peak tops differed between each sample: 48.5, 51.1, and 53.2 nm for FPS_48_(80/20), FPS_54_(80/20), and FPS_57_(80/20), respectively. These diameters, calculated using BJH theory, closely align with the SEM results (Figure 2). This inconsistency may arise from the broadening of the peaks in the pore diameter distribution (Figure 2(b)). Furthermore, the larger mesopores were derived from the PS blocks. The molecular weights of the PS blocks were 47.5, 53.9, and 57.0 kg mol^–1^ for FPS_48_(80/20), FPS_54_(80/20), and FPS_57_(80/20), respectively. Consequently, the diameters of the larger mesopores differed for each sample. Bimodal MPCs are expected to enable simultaneous multifunctionality due to their different pore sizes. For example, larger mesopores are anticipated to facilitate mass and ion transport, while smaller mesopores are expected to serve as reaction sites. This dual pore structure is therefore thought to allow for the efficient transport of various molecules.

In this study, we demonstrated the fabrication of nitrogen-doped bimodal MPCs in mesopore regions using organic–organic hybrids with FPS triblock terpolymers and resol. Blend films of the FPS triblock terpolymers and resol formed hexagonally packed PS cylinders surrounded by six-coordinated PTFEMA spheres; the spherical domains were aligned along the PS cylinders in the P4VP/resol matrix domains. This structure was obtained because of the low affinity of PS for DMF despite PS exhibiting the highest volume fraction. After carbonization, the PTFEMA and PS domains were removed, and bimodal MPCs containing smaller spherical mesopores, larger cylindrical mesopores, and nitrogen-doped carbon walls were fabricated. The pore sizes of the MPCs were estimated to be approximately 10 and 30 nm in diameter by using SAXS and SEM techniques, respectively. In addition, larger pore sizes could be changed according to the molecular weight of the PS blocks. Furthermore, nitrogen adsorption measurements revealed a large specific surface area (up to 700 m^2^ g^–1^) and an evident bimodal distribution. ABC triblock terpolymers with a hydrophilic middle B block can serve as a candidate template for highly ordered bimodal MPCs and exhibit high potential for adsorption, oxygen reduction reactions, drug delivery, and other applications.

## Supporting Information Available

Sample preparation procedures and chemical and structural analyses: ^1^H NMR, SEC, and elemental analysis (PDF)

## References

[ref1] Kennedy L. J.; Vijaya J. J.; Kayalvizhi K.; Sekaran G. Adsorption of Phenol from Aqueous Solutions Using Mesoporous Carbon Prepared by Two-Stage Process. Chem. Eng. J. 2007, 132, 279–287. 10.1016/j.cej.2007.01.009.

[ref2] Ji L. L.; Liu F. L.; Xu Z. Y.; Zheng S. R.; Zhu D. Q. Adsorption of Pharmaceutical Antibiotics on Template-Synthesized Ordered Micro- and Mesoporous Carbons. Environ. Sci. Technol. 2010, 44, 3116–3122. 10.1021/es903716s. 20201519

[ref3] Hartmann M.; Vinu A.; Chandrasekar G. Adsorption of Vitamin E on Mesoporous Carbon Molecular Sieves. Chem. Mater. 2005, 17, 829–833. 10.1021/cm048564f.

[ref4] Feng D.; Lv Y. Y.; Wu Z. X.; Dou Y. Q.; Han L.; Sun Z. K.; Xia Y. Y.; Zheng G. F.; Zhao D. Y. Free-standing Mesoporous Carbon Thin Films with Highly Ordered Pore Architectures for Nanodevices. J. Am. Chem. Soc. 2011, 133, 15148–15156. 10.1021/ja2056227. 21854032

[ref5] Yuan B.; Wu X. F.; Chen Y. X.; Huang J. H.; Luo H. M.; Deng S. G. Adsorption of CO_2_, CH_4_, and N_2_ on Ordered Mesoporous Carbon: Approach for Greenhouse Gases Capture and Biogas Upgrading. Environ. Sci. Technol. 2013, 47, 5474–5480. 10.1021/es4000643. 23688273

[ref6] Zhou M.; Ding J.; Guo L. P.; Shang Q. K. Electrochemical Behavior of L-cysteine and Its Detection at Ordered Mesoporous Carbon-Modified Glassy Carbon Electrode. Anal. Chem. 2007, 79, 5328–5335. 10.1021/ac0703707. 17555298

[ref7] Huang X.; Wu S. S.; Du X. Z. Gated Mesoporous Carbon Nanoparticles as Drug Delivery System for Stimuli-Responsive Controlled Release. Carbon. 2016, 101, 135–142. 10.1016/j.carbon.2016.01.094.

[ref8] Saha D.; Warren K. E.; Naskar A. K. Soft-Templated Mesoporous Carbons as Potential Materials for Oral Drug Delivery. Carbon. 2014, 71, 47–57. 10.1016/j.carbon.2014.01.005.

[ref9] Su F. B.; Zeng J. H.; Bao X. Y.; Yu Y. S.; Lee J. Y.; Zhao X. S. Preparation and Characterization of Highly Ordered Graphitic Mesoporous Carbon as a Pt Catalyst Support for Direct Methanol Fuel Cells. Chem. Mater. 2005, 17, 3960–3967. 10.1021/cm0502222.

[ref10] Guo L. M.; Wang X. T.; Wang Y. Facile Synthesis of Bimodal Nanoporous Carbons by Templating Selective Swelling-Induced Mesoporous Block Copolymers. Chem. Eng. J. 2017, 313, 1295–1301. 10.1016/j.cej.2016.11.028.

[ref11] Liu Y. Y.; Zeng G. M.; Tang L.; Cai Y.; Pang Y.; Zhang Y.; Yang G.; Zhou Y. Y.; He X. X.; He Y. Highly Effective Adsorption of Cationic and Anionic Dyes on Magnetic Fe/Ni Nanoparticles Doped Bimodal Mesoporous Carbon. J. Colloid Interface Sci. 2015, 448, 451–459. 10.1016/j.jcis.2015.02.037. 25765736

[ref12] Zhuang X.; Wan Y.; Feng C. M.; Shen Y.; Zhao D. Y. Highly Efficient Adsorption of Bulky Dye Molecules in Wastewater on Ordered Mesoporous Carbons. Chem. Mater. 2009, 21, 706–716. 10.1021/cm8028577.

[ref13] Montiel G.; Fuentes-Quezada E.; Bruno M. M.; Corti H. R.; Viva F. A. Effect of Bimodal Mesoporous Carbon as PtRu Catalyst Support for Direct Methanol Fuel Cells. RSC Adv. 2020, 10, 30631–30639. 10.1039/D0RA05676F. 35516039 PMC9056354

[ref14] Mandlmeier B.; Niedermayer S.; Schmidt A.; Schuster J.; Bein T. Lipid-Bilayer Coated Nanosized Bimodal Mesoporous Carbon Spheres for Controlled Release Applications. J. Mater. Chem. B 2015, 3, 9323–9329. 10.1039/C5TB01635E. 32262931

[ref15] Deng Y. H.; Liu C.; Yu T.; Liu F.; Zhang F. Q.; Wan Y.; Zhang L. J.; Wang C. C.; Tu B.; Webley P. A.; et al. Facile Synthesis of Hierarchically Porous Carbons from Dual Colloidal Crystal/Block Copolymer Template Approach. Chem. Mater. 2007, 19, 3271–3277. 10.1021/cm070600y.

[ref16] Kresge C. T.; Leonowicz M. E.; Roth W. J.; Vartuli J. C.; Beck J. S. Ordered Mesoporous Mplecular-Sieves Synthesized by a Liquid-Crystal Template Mechanism. Nature. 1992, 359, 710–712. 10.1038/359710a0.

[ref17] Beck J. S.; Vartuli J. C.; Roth W. J.; Leonowicz M. E.; Kresge C. T.; Schmitt K. D.; Chu C. T. W.; Olson D. H.; Sheppard E. W.; McCullen S. B.; et al. A New Family of Mesoporous Molecular Sieves Prepared with Liquid Crystal Templates. J. Am. Chem. Soc. 1992, 114, 10834–10843. 10.1021/ja00053a020.

[ref18] Huo Q. S.; Margolese D. I.; Ciesla U.; Feng P. Y.; Gier T. E.; Sieger P.; Leon R.; Petroff P. M.; Schuth F.; Stucky G. D. Generalized Synthesis of Periodic Surfactant/Inorganic Composite Materials. Nature. 1994, 368, 317–321. 10.1038/368317a0.

[ref19] Zhao D. Y.; Huo Q. S.; Feng J. L.; Chmelka B. F.; Stucky G. D. Nonionic Triblock and Star Diblock Copolymer and Oligomeric Surfactant Syntheses of Highly Ordered, Hydrothermally Stable, Mesoporous Silica Structures. J. Am. Chem. Soc. 1998, 120, 6024–6036. 10.1021/ja974025i.

[ref20] Ryoo R.; Joo S. H.; Jun S. Synthesis of Highly Ordered Carbon Molecular Sieves via Template-Mediated Structural Transformation. J. Phys. Chem. B 1999, 103, 7743–7746. 10.1021/jp991673a.

[ref21] Ryoo R.; Joo S. H. Nanostructured Carbon Materials Synthesized from Mesoporous Silica Crystals by Replication. Studies in Surface Science and Catalysis 2004, 148, 241–260. 10.1016/S0167-2991(04)80200-3.

[ref22] Liang C. D.; Dai S. Synthesis of Mesoporous Carbon Materials via Enhanced Hydrogen-Bonding Interaction. J. Am. Chem. Soc. 2006, 128, 5316–5317. 10.1021/ja060242k. 16620083

[ref23] Fang Y.; Gu D.; Zou Y.; Wu Z. X.; Li F. Y.; Che R. C.; Deng Y. H.; Tu B.; Zhao D. Y. A Low-Concentration Hydrothermal Synthesis of Biocompatible Ordered Mesoporous Carbon Nanospheres with Tunable and Uniform Size. Angew. Chem., Int. Ed. Engl. 2010, 49, 7987–7991. 10.1002/anie.201002849. 20839199

[ref24] Meng Y.; Gu D.; Zhang F. Q.; Shi Y. F.; Cheng L.; Feng D.; Wu Z. X.; Chen Z. X.; Wan Y.; Stein A.; et al. A Family of Highly Ordered Mesoporous Polymer Resin and Carbon Structures from Organic-Organic Self-Assembly. Chem. Mater. 2006, 18, 4447–4464. 10.1021/cm060921u.

[ref25] Li M.; Xue J. M. Ordered Mesoporous Carbon Nanoparticles with Well-Controlled Morphologies from Sphere to Rod via a Soft-Template Route. J. Colloid Interface Sci. 2012, 377, 169–175. 10.1016/j.jcis.2012.03.085. 22542324

[ref26] Huang Y.; Cai H. Q.; Yu T.; Sun X. L.; Tu B.; Zhao D. Y. Highly Ordered Mesoporous Carbonaceous Frameworks from a Template of a Mixed Amphiphilic Triblock-Copolymer System of PEO-PPO-PEO and Reverse PPO-PEO-PPO. Chem.–Asian J. 2007, 2, 1282–1289. 10.1002/asia.200700173. 17685375

[ref27] Qian X. F.; Li H. X.; Wan Y. Structure Design of Mesoporous Carbons by Blending PEO-PPO-PEO-Type and PPO-PEO-PPO-Type Amphiphilic Block Copolymers in Organic-Organic Self-Assembly. Microporous Mesoporous Mater. 2011, 141, 26–37. 10.1016/j.micromeso.2009.11.012.

[ref28] Liang C. D.; Hong K. L.; Guiochon G. A.; Mays J. W.; Dai S. Synthesis of a Large-Scale Highly Ordered Porous Carbon Film by Self-Assembly of Block Copolymers. Angew. Chem., Int. Ed. Engl. 2004, 43, 5785–5789. 10.1002/anie.200461051. 15523736

[ref29] Cao S. B.; Qu T.; Li Y. Y.; Zhang A.; Xue L. F.; Zhao Y. B.; Zheng L. R.; Chen A. H.; Shui J. L. Electrocatalytically Active Hollow Carbon Nanospheres Derived from PS-*b*-P4VP Micelles. Part. Part. Syst. Charact. 2018, 35, 9 10.1002/ppsc.201700404.

[ref30] Fei H. F.; Li W. H.; Bhardwaj A.; Nuguri S.; Ribbe A.; Watkins J. J. Ordered Nanoporous Carbons with Broadly Tunable Pore Size Using Bottlebrush Block Copolymer Templates. J. Am. Chem. Soc. 2019, 141, 17006–17014. 10.1021/jacs.9b09572. 31577903

[ref31] Werner J. G.; Hoheisel T. N.; Wiesner U. Synthesis and Characterization of Gyroidal Mesoporous Carbons and Carbon Monoliths with Tunable Ultralarge Pore Size. ACS Nano 2014, 8, 731–743. 10.1021/nn405392t. 24328285

[ref32] Werner J. G.; Johnson S. S.; Vijay V.; Wiesner U. Carbon-Sulfur Composites from Cylindrical and Gyroidal Mesoporous Carbons with Tunable Properties in Lithium-Sulfur Batteries. Chem. Mater. 2015, 27, 3349–3357. 10.1021/acs.chemmater.5b00500.

[ref33] Zhang Q.; Matsuoka F.; Suh H. S.; Beaucage P. A.; Xiong S. S.; Smilgies D. M.; Tan K. W.; Werner J. G.; Nealey P. F.; Wiesner U. B. Pathways to Mesoporous Resin/Carbon Thin Films with Alternating Gyroid Morphology. ACS Nano 2018, 12, 347–358. 10.1021/acsnano.7b06436. 29236479

[ref34] Hesse S. A.; Beaucage P. A.; Smilgies D. M.; Wiesner U. Structurally Asymmetric Porous Carbon Materials with Ordered Top Surface Layers from Nonequilibrium Block Copolymer Self-Assembly. Macromolecules. 2021, 54, 2979–2991. 10.1021/acs.macromol.0c02720.

[ref35] Hesse S. A.; Werner J. G.; Wiesner U. One-Pot Synthesis of Hierarchically Macro- and Mesoporous Carbon Materials with Graded Porosity. ACS Macro Lett. 2015, 4, 477–482. 10.1021/acsmacrolett.5b00095. 35596287

[ref36] Deng G. D.; Qiang Z.; Lecorchick W.; Cavicchi K. A.; Vogt B. D. Nanoporous Nonwoven Fibril-Like Morphology by Cooperative Self-Assembly of Poly(Ethylene Oxide)-Block-Poly(Ethyl Acrylate)-*Block*-Polystyrene and Phenolic Resin. Langmuir. 2014, 30, 2530–2540. 10.1021/la404964c. 24548298

[ref37] Li J.-G.; Lin R.-B.; Kuo S.-W. Hierarchical Mesoporous Silica Fabricated from an ABC Triblock Terpolymer as a Single Template. Macromol. Rap. Commun. 2012, 33 (8), 678–682. 10.1002/marc.201100857. 22354763

[ref38] Ahn S.; Kwak J.; Choi C.; Seo Y.; Kim J. K.; Lee B. Gyroid Structures at Highly Asymmetric Volume Fractions by Blending of ABC Triblock Terpolymer and AB Diblock Copolymer. Macromolecules 2017, 50, 9008–9014. 10.1021/acs.macromol.7b01734.

[ref39] Miyamori Y.; Tong L.; Nabae Y.; Hatakeyama-Sato K.; Hayakawa T. Core-Shell Double Gyroids Directed by Selective Solvation for ABC Triblock Terpolymers. Macromol. Rap. Commun. 2024, 45 (14), 8 10.1002/marc.202400093. 38639102

