# Supplementary material for: Highly Ordered Bimodal Mesoporous Carbon from ABC Triblock Terpolymers with Phenolic Resol
Source: ACS Macro Lett. 2024 Dec 5;13(12):1698–703. doi: 10.1021/acsmacrolett.4c00651 (PMC11656712; doi:10.1021/acsmacrolett.4c00651)
Supplement: Supplementary file 1 — mz4c00651_si_001.pdf [file mz4c00651_si_001.pdf]

Highly ordered bimodal mesoporous carbon from ABC triblock terpolymers with phenolic resol

Yuta Miyamori[1], Youngwon Kong[1], Yuta Nabae[1], Kan Hatakeyama-Sato[1], and Teruaki Hayakawa[1]*

Department of Materials Science and Engineering, School of Materials and Chemical Technology, Institute of Science Tokyo, 2-12-1 S8-36 Ookayama, Meguro-ku, Tokyo, 152-8552 Japan

Synthesis of poly(2,2,2-trifluoroethyl methacrylate) (PTFEMA)-*b*-poly(4-vinylpyridine) (P4VP)-*b*-polystyrene (PS) (FPS triblock terpolymer)

Synthesizing of a PTFEMA homopolymer

A PTFEMA homopolymer was synthesized 2-cyano-2-propyl dodecyl trithiocarbonate (CPDT) as a chain transfer agent (CTA). CPDT (570 mg, 1.6 mmol), 2,2,2-trifluoroethyl methacrylate (9.9 g, 58.9 mmol), and AIBN (0.32 mmol (0.2 eq. for CPDT) were mixed in a schlenk flask. Freeze and pump cycles were conducted 5 times, and Ar gas was purged into schlenk flasks. The solution was heated at 70 °C for 4 hours. After cooling at room temperature, the reactant was diluted by THF and reprecipitated in *n*-hexane.

Synthesizing of an FP diblock copolymer

An FP diblock copolymer was synthesized using a PTFEMA homopolymer as a macro-CTA. A PTFEMA homopolymer (13.4 kg mol^{-1}, D = 1.27, 2.0 g, 0.15 mmol), 4-vinylpyridine (12.8 g, 121.6 mmol), THF which has an equal volume of 4-vinylpyridine, and AIBN (0.03 mmol (0.2 eq. for PTFEMA homopolymer) were mixed in a schlenk flask. Freeze and pump cycles were conducted 5 times, and Ar gas was purged into schlenk flasks. The solution was heated at 70 °C for 6 hours. After cooling at room temperature, the reactant was diluted by THF and reprecipitated in *n*-hexane. An FP diblock copolymer was obtained as yellow powders (M_n = 27.8 kg mol^{-1}, φ_F:φ_P = 0.42:0.58, D = 1.09).

Synthesizing of FPS triblock terpolymers

FPS triblock terpolymers were synthesized using an FP diblock copolymer as a macro-CTA. FP diblock copolymer (M_n = 27.8 kg mol^{-1}, φ_F:φ_P = 0.42:0.58, $Đ$ = 1.09, 1.9 g, 0.07 mmol), styrene (24.8 g, 196.7 mmol), DMF which has an equal volume of styrene, and AIBN (0.014 mmol (0.2 eq. for FP diblock copolymer)) were mixed. The solution was divided into three schlenk flasks. Freeze and pump cycles were conducted 5 times, and Ar gas was purged into schlenk flasks. The solution was heated at 70 °C for 24, 36, and 48 hours. After cooling at room temperature, the reactant was diluted by CH_2Cl_2 and reprecipitated in *n*-hexane. Consequently, FPS_{48}, FPS_{54}, and FPS_{57} were obtained as yellow powders (molecular characteristics are shown in Table 1). After synthesizing FPS triblock terpolymer, we conducted Soxhlet extraction to remove byproduct polystyrene homopolymer using cyclohexane at 120 °C.

Sample preparation

Preparation of composite films

5 wt% DMF solutions of the FPS triblock terpolymer and resol were mixed in 50 mL vials. The mixed solutions were dried under reduced pressure (10 kPa) on a hot plate at 30 °C for one week. After visual confirmation that the bulk films were perfectly dried, the composite films were vacuum-dried at 30 °C. The dried films were then heated at 100 °C for 24 hours under vacuum to crosslink the resol. Consequently, FPS_{48}(80/20), FPS_{54}(80/20), and FPS_{57}(80/20) were acquired at 490.4 mg, 479.2 mg, and 481.8 mg, respectively.

Preparation of mesoporous carbons (MPCs)

MPCs were obtained via the thermal treatment of composite films. Composite films were heated at a rate of 1 °C min^{-1} up to 600 °C, 10 °C min^{-1} from 600 to 900 °C, and then held at 900 °C for 1 hour under a nitrogen atmosphere. As a result of the thermal treatment, it was found that the weight of composite films decreased by approximately 86 wt%.

Chemical characterization

^{1}H NMR spectra were recorded on a JEOL JNM-ECS400 (400 MHz) spectrometer using

$CDCl_3$. Size exclusion chromatography (SEC) was performed by ASAHI TECHNION Viscotek GPCmax VE-2001 equipped with a Viscotek TDA 302 and a Shodex column LF 804×2) with 50 mmol L^{-1} LiBr in DMF at a flow rate of 1.0 mL min^{-1} at 40 °C. Elemental analysis was carried out CHN analyzer (JM10 provided by J-Science), ion chromatograph system (ICS1100 provided by Thermo Fisher Scientific) for quantifying fluorine, and Vario micro cube provided by Elementar for quantifying oxygen.

Structural analysis

Transmission electron microscopy (TEM) observation was performed by H7650 instrument (Hitachi High-Technologies) operating at 100 kV to reveal the microphase-separated structures of composite films. Samples (approximately 70 nm thickness) were prepared on an EM UC7/FC7 (Leica Microsystems) using a diamond knife at room temperature. Electron scattering contrast was achieved by preferentially staining the P4VP domains by exposing the cut specimens to I_2. In contrast, scanning electron microscopy (SEM) observation was performed by JSM-7500F instruments (JEOL) to acquire structural information regarding MPCs. SAXS measurements were conducted using an X-ray beam on the bulk films at SPring-8 BL40B2 or in-house SAXS (Bruker SAXS NanoSTAR instrument, output: 50 kV, 50 mA). In SPring-8 BL40B2, the sample-to-detector distance was approximately 2000 mm, and the X-ray wavenumber was approximately 1.95 □ (CuKα irradiation). Nitrogen adsorption measurement was performed using Belsorp-mini □ (Bel Japan Inc.) and analyzed by Brunauer-Emmett-Teller (BET) and Barrett-Joyner-Halenda (BJH) theory to reveal the specific surface area and pore diameter distribution of MPCs. MPCs were thermally treated at 350 °C for 1.5 hours under a vacuum to eliminate impurities like water before nitrogen adsorption measurement.

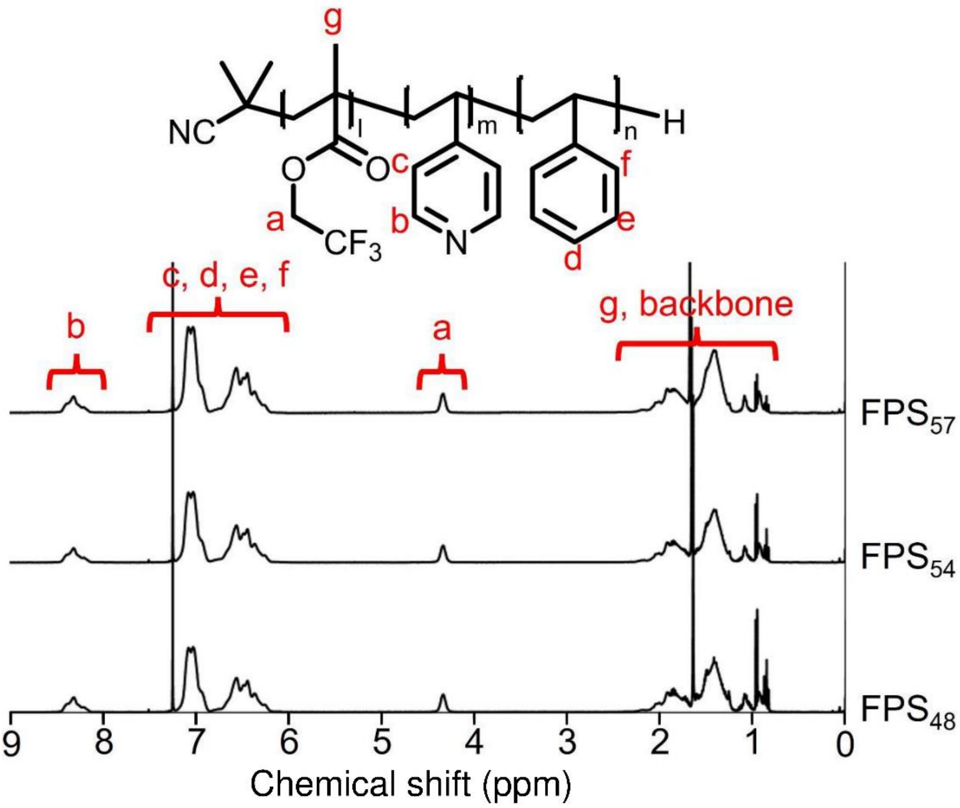

Figure S1 ^{1}H NMR spectra of FPS_{48}, FPS_{54}, and FPS_{57}

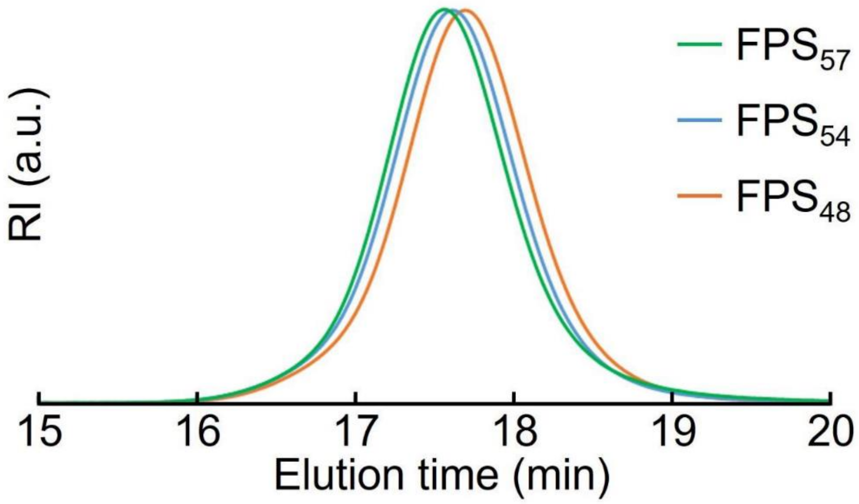

Figure S2 SEC chromatograms of FPS_{48}, FPS_{54}, and FPS_{57}.

Table S1 The result of elemental analysis

Sample	Content (wt%)				
	H	C	N	O	F
FPS_{48}(80/20)	0.8	92.9	1.1	5.2	NA
FPS_{54}(80/20)	1.2	86.8	0.9	11.1	NA
FPS_{57}(80/20)	1.3	86.0	1.0	11.7	NA

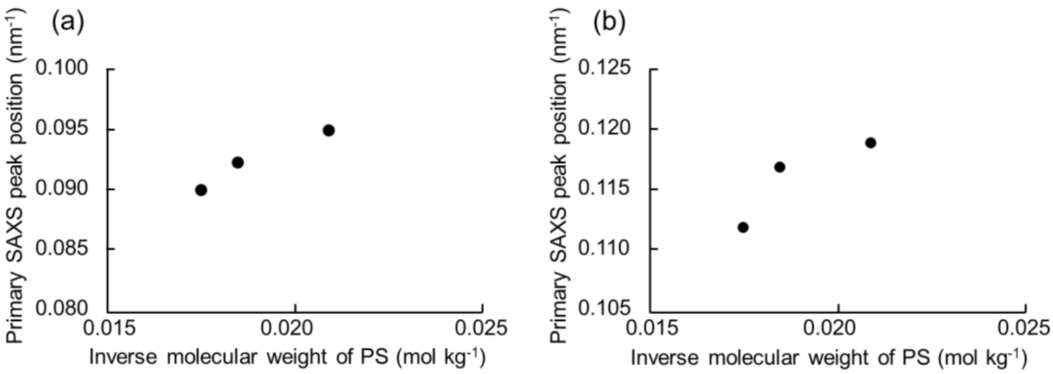

Figure S3 Correlation between the primary SAXS peak and the molecular weights of PS blocks (a) before and (b) after carbonization.
